# Kombucha-Derived Bacterial Cellulose Nanowhisker-Reinforced Electroblown Gelatin/PVA Nanofibrous Mats as Candidate Materials for Sustainable Food Packaging

**DOI:** 10.3390/polym18141764

**Published:** 2026-07-19

**Authors:** Salih Birhanu Ahmed, Andinet Kumella Eticha, Harun Cug, Nurcan Dogan, Cemhan Dogan, Sedef Sismanoglu, Nagham Elberishy, Yasin Akgul, Islam Shyha

**Affiliations:** 1 Department of Mechanical Engineering, Faculty of Engineering and Natural Sciences, Karabuk University, 78050 Karabuk, Türkiye; salihbirhanu@gmail.com (S.B.A.); hcug@karabuk.edu.tr (H.C.); 2 School of Mechanical and Industrial Engineering, College of Technology and Built Environment, Addis Ababa University, Addis Ababa 1000, Ethiopia; andinetkumella48@gmail.com; 3 Department of Food Technology, Bogazliyan Vocational School, Yozgat Bozok University, 66400 Yozgat, Türkiye; nurcan.dogan@bozok.edu.tr (N.D.); cemhan.dogan@bozok.edu.tr (C.D.); 4 Department of Chemistry, Faculty of Engineering and Natural Sciences, Karabuk University, 78050 Karabuk, Türkiye; sedefsismanoglu@karabuk.edu.tr; 5 Production Engineering Department, Faculty of Engineering, Alexandria University, Alexandria 21544, Egypt; n.elberishy@ubt.edu.sa; 6 Department of Industrial Engineering, University of Business and Technology, Jeddah 23847, Saudi Arabia; 7 Department of Biomedical Engineering, Faculty of Engineering and Natural Sciences, Karabuk University, 78050 Karabuk, Türkiye; 8 School of Computing Engineering and the Built Environment, Edinburgh Napier University, Edinburgh EH14 1DJ, UK

**Keywords:** kombucha-derived bacterial cellulose, bacterial cellulose nanowhiskers, gelatin/PVA, electroblowing, biodegradable packaging, nanofibrous mats

## Abstract

The growing demand for sustainable food packaging materials has accelerated the development of biodegradable alternatives to conventional petroleum-based plastic. This study investigates the reinforcement of electroblown gelatin/polyvinyl alcohol (G-PVA) nanofibrous mats with kombucha-derived bacterial cellulose nanowhiskers (BCNWs) to enhance their mechanical, thermal, and surface properties while promoting the valorization of symbiotic culture of bacteria and yeast (SCOBY) waste. BCNWs were incorporated into the G-PVA matrix at different loadings, and the resulting nanocomposites were characterized using scanning electron microscopy (SEM), Fourier-transform infrared spectroscopy (FTIR), X-ray diffraction (XRD), thermogravimetric analysis (TGA), tensile testing, and water contact angle measurements. The addition of BCNW significantly improved the tensile strength of the nanofibrous mats, with a maximum increase about 120% compared with neat G-PVA nanofibers. SEM images revealed uniform, bead-free fiber structures at appropriate BCNW concentrations, while FTIR and XRD analyses confirmed effective interactions between the nanowhiskers and the polymer matrix. TGA results indicated enhanced thermal stability at moderate BCNW loadings, whereas excessive BCNW content promoted agglomeration and reduced thermal resistance. Furthermore, the incorporation of BCNWs increased the water contact angle to 144.11 ± 1.45°, demonstrating improved surface hydrophobicity. Overall, kombucha-derived BCNWs effectively reinforced electroblown G-PVA nanofibers, producing biodegradable nanocomposite mats with improved performance and strong potential for sustainable food packaging applications.

## 1. Introduction

Growing environmental concerns associated with synthetic, non-degradable packaging materials have spurred extensive research into bio-based, sustainable alternatives for the food packaging industry [1]. Traditional petroleum-based packaging contributes significantly to environmental pollution, with millions of tons of waste entering the oceans annually, posing a severe threat to marine ecosystems [2]. This pressing issue has motivated scientists and industries to explore biodegradable, renewable materials to minimize the ecological footprint of food packaging [3]. Among the various biopolymers investigated, bacterial cellulose, gelatin, and polyvinyl alcohol have emerged as promising candidates due to their inherent properties, including biodegradability, biocompatibility, and non-toxicity [4,5]. The shift towards biodegradable food packaging underscores growing concern about the environmental impact of conventional petrochemical-based packaging, driving the search for novel biopolymers with enhanced functionality. The use of biopolymers derived from readily available polysaccharides, proteins, and lipids presents a promising route to biodegradable packaging materials [6]. This strategy supports a circular economy by converting an abundant by-product into value-added materials.

The development of sustainable food packaging demands a holistic perspective that considers both material performance and environmental impact across the entire life cycle. In this context, active packaging, which incorporates functional agents that inhibit microbial growth and enhance food preservation, is being explored using biodegradable polymers [7,8]. Gelatin, a protein derived from collagen, is an attractive biopolymer for food packaging applications due to its excellent film-forming ability, biodegradability, and edibility. Gelatin’s ability to form transparent, flexible films makes it suitable for food packaging, though its poor mechanical and water-vapor barrier properties limit its use [9]. Polyvinyl alcohol, a synthetic polymer, is biodegradable under specific conditions and has good film-forming and gas barrier properties. Blending gelatin and polyvinyl alcohol can leverage the complementary properties of both polymers, resulting in a composite material with improved mechanical strength, flexibility, and barrier properties.

The use of nanofibers in active food packaging is gaining attention due to their unique properties, which enhance food quality, preservation, and safety [10]. These nanofibers offer high surface area, porosity, and the ability to incorporate bioactive compounds, making them ideal for extending the shelf life of food products [11]. The fabrication of nanofiber mats through electroblowing offers a versatile approach to produce high-surface-area materials with controlled morphology. Electroblowing is a technique that uses an electrical charge and pressurized air to draw fibers from a liquid. Electro-spun nanofiber mats have found applications in various fields, including active food packaging, drug delivery, and filtration, owing to their high porosity, interconnected structure, and tunable properties [12]. For example, in a study by Merkle et al. [13], the addition of PVA raised gelatin’s Young’s modulus from 21.52 ± 4.15 MPa to 168.6 ± 36.5 MPa, and its tensile strength from 0.48 ± 0.02 MPa to 5.42 ± 1.95 MPa. Similarly, Akhouy et al. [14] reported that blending PVA into a gelatin–chitosan matrix improved tensile strength from 0.8345 ± 0.322 MPa to 1.3672 ± 0.226 MPa, underscoring the effectiveness of PVA in reinforcing gelatin-based fibers.

The integration of nanomaterials, particularly nanocellulose, into biopolymer matrices has attracted considerable attention as a strategy to overcome the limitations of biopolymers, such as their weak mechanical and barrier properties [15]. Nanocellulose, derived from plant or bacterial sources, possesses exceptional mechanical strength, high surface area, and biodegradability, making it an ideal reinforcing agent for biopolymer composites [1,16,17]. Cellulose, a sustainable and abundantly available biopolymer derived from natural sources such as plants, algae, fungi, and bacteria, stands out as an ornate biopolymer [18].

Bacterial cellulose, synthesized by bacteria such as *Komagataeibacter xylinus*, offers distinct advantages over plant-derived cellulose, including higher purity, crystallinity, and mechanical strength [19]. In recent years, kombucha fermentation has emerged as a sustainable source of bacterial cellulose. During fermentation of kombucha, a symbiotic culture of bacteria and yeast (SCOBY) forms as thick cellulose pellicles, which are often discarded as byproducts [20]. Valorizing SCOBY as a feedstock for bacterial cellulose nanowhiskers (BCNWs) not only provides a cost-effective and renewable raw material but also transforms a fermentation waste into a high-value reinforcing agent for biopolymer composites [21].

Although bacterial cellulose, gelatin, and polyvinyl alcohol (PVA) have been extensively investigated for food packaging applications due to their biodegradability, film-forming ability, and biocompatibility [22], recent studies have emphasized the need for more sustainable feedstocks and enhanced multifunctionality of these materials. Kombucha-derived bacterial cellulose has emerged as a promising alternative because it is generated as a low-cost fermentation byproduct with high purity, crystallinity, and mechanical strength, making it highly suitable for sustainable material development. In this study, kombucha-derived bacterial cellulose nanowhiskers (BCNWs) were incorporated into electroblown gelatin/PVA nanofibrous mats, introducing a novel reinforcement strategy that combines waste valorization with nanostructural enhancement. Unlike previous BC-based packaging systems, this work systematically correlates BCNW loading with the morphological, thermal, mechanical, and wettability properties of electroblown nanofibers, providing new insights into structure–property relationships for eco-friendly food packaging applications.

This study investigates the fabrication and characterization of gelatin–polyvinyl alcohol (G–PVA) based on one-to-one ratio nanofibrous mats reinforced with BCNWs at varying concentrations (2%, 4%, 6%, and 10%) as potential sustainable materials for food packaging applications. The gelatin/PVA blend ratio used in this study (1:1, *w*/*w*) was selected based on previous reports demonstrating that this composition provides a favorable balance between the mechanical strength, spinnability, and biodegradability of the resulting nanofibers. Gelatin contributes excellent biocompatibility and biodegradability but exhibits poor mechanical strength and moisture resistance, whereas PVA enhances solution spinnability, fiber uniformity, and structural stability. A 1:1 blend has been reported to produce homogeneous nanofibrous structures with improved mechanical performance and reduced bead formation compared with formulations rich in either gelatin or PVA [23]. Therefore, this ratio was adopted as the baseline formulation to investigate the reinforcing effect of kombucha-derived bacterial cellulose nanowhiskers (BCNWs), while optimization of the polymer blend composition was considered beyond the scope of the present study. Although gelatin is well-suited for food-contact applications, its practical use is often limited by its low mechanical strength and pronounced hydrophilicity, which stem from its relatively short polymer chains and restricted chain mobility. To address these limitations, gelatin was blended with polyvinyl alcohol (PVA), a biodegradable polymer known for its high flexibility, good compatibility, and excellent gas barrier properties. The incorporation of BCNWs further aims to reinforce the polymer matrix and improve overall structural performance. By adjusting the composition and processing parameters, the properties of the electroblown nanofibers can be effectively tailored for targeted applications, including sustainable food packaging.

## 2. Materials and Methods

### 2.1. Materials

Type A bovine gelatin with a Bloom strength of 250–270, in powdered form and derived from bovine skin, was kindly donated by Halavet Gida LLC, Istanbul, Turkey. Merck, Darmstadt, Germany, supplied 100% pure anhydrous acetic acid. PVA was donated by ZAG Kimya (Istanbul, Turkey) and has a purity of 87.8%, a density of 1.2–1.3 g/cm^3^ at 20 °C, and a melting range of 160–240 °C. Ethanol (>95% purity) was supplied by TEKKİM Chemicals (Istanbul, Türkiye), with a density of 0.801–0.805 g/cm^3^ at 20 °C and a vapor pressure of 59 KPa. Ultra-distilled water was used as the solvent.

### 2.2. Synthesis and Characterization of BCNW

BCNWs were extracted from the dried pulp waste of kombucha tea, namely, kombucha-derived bacterial cellulose biomass, which represents a sustainable and abundant cellulose source. Initially, the kombucha pulp was thoroughly washed with distilled water to remove residual sugars, soluble fermentation by-products, proteins, and microbial residues. The cleaned biomass was then subjected to alkaline purification by immersion in 1 M sodium hydroxide (NaOH) at 80 °C for 2 h to remove non-cellulosic impurities. After alkaline treatment, the cellulose-rich material was repeatedly rinsed with distilled water until a neutral pH was obtained. To isolate bacterial cellulose nanowhiskers, the purified bacterial cellulose was hydrolyzed using 45% H_2_SO_4_ at 50 °C for 45 min under continuous stirring. This acid hydrolysis process selectively removes the amorphous regions of cellulose while preserving the crystalline domains, thereby yielding nanoscale cellulose whiskers. After hydrolysis, the reaction was immediately quenched by dilution with ice-cold distilled water. The resulting suspension was subjected to repeated centrifugation cycles at 10,000 rpm for 10 min, followed by washing with distilled water to remove excess sulfuric acid and soluble hydrolysis byproducts. To further ensure the removal of residual free acid, the BCNW suspension was dialyzed against distilled water for 5 days using a cellulose dialysis membrane with a molecular weight cut-off of 12–14 kDa. The dialysis water was regularly replaced, and dialysis was continued until the external dialysis medium reached a stable neutral pH. The attainment of a constant neutral pH was considered practical evidence for the removal of residual free acid from the BCNW suspension. It should be noted that this purification step confirms the removal of free acid residues, whereas sulfate ester groups that may be introduced onto the cellulose surface during sulfuric acid hydrolysis are considered surface functional groups of the resulting BCNWs rather than residual free acid. The final colloidal BCNW suspension was stored at 4 °C and briefly sonicated before use in nanofiber fabrication. The morphology and size of BCNWs were examined using transmission electron microscopy (TEM; JEOL JEM-1220, Tokyo, Japan) operated at 80 kV. For TEM analysis, a drop of the diluted BCNW suspension was deposited onto a carbon-coated copper grid and dried at room temperature before imaging.

### 2.3. Preparation of Solutions

G–PVA solution was prepared by dissolving gelatin and PVA separately, then blending them in a 1:1 ratio. The gelatin solution was prepared by dissolving 5.8% gelatin in 29% acetic acid and stirring for 4 h. Similarly, PVA solution was prepared by dissolving 5.8% PVA in a co-solvent mixture of 46% distilled water and 13.0% ethanol (Eth), followed by continuous stirring for 4 h.

After the individual polymer solutions were prepared, BCNWs were incorporated into the total polymer content at 2, 4, 6, and 10 wt.% loadings, yielding the G-PVA-2BCNW, G-PVA-4BCNW, G-PVA-6BCNW, and G-PVA-10BCNW formulations. Mixtures were then stirred for an additional 4 h to promote complete dispersion and ensure solution homogeneity.

### 2.4. Fiber Production Process

**Figure 1:**
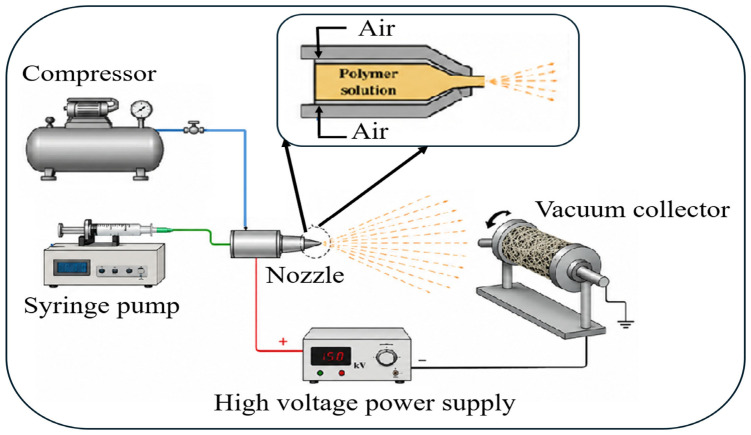
Schematic illustration of the electroblowing fiber production technique.

Nanofibrous mats were fabricated using the electroblowing technique. Solutions were delivered through a nozzle via a 20 mL syringe mounted on a syringe pump. The nozzle and the system frame were connected to a high-voltage power supply to establish the required electric field. A schematic illustration of the electroblowing setup is shown in Figure 1.

Fiber formation was achieved by maintaining a constant air pressure of 1.5 bar and an applied voltage of 15 kV, which provided sufficient shear force for stable jet formation. The solution was fed at a controlled rate of 15 mL h^−1^. The resulting nanofibers were collected on a rotating collector positioned 35 cm from the nozzle. After collection, nanofibrous mats were thermally treated at 170 °C for 2 h to improve structural stability.

### 2.5. Characterization of Nanofibers

#### 2.5.1. Scanning Electron Microscope (SEM)

A Carl Zeiss Ultra Plus field-emission scanning electron microscope (FE-SEM) was employed to examine the microstructure of the fibrous mats. Fiber diameters and size distributions were quantified using ImageJ (version 1.53e), with 100 measurements taken from randomly selected micrographs for each sample to ensure statistical reliability.

#### 2.5.2. Fourier Transform Infrared (FTIR) Spectroscopy

FTIR spectra of the fibrous mats were recorded using a Bruker ALPHA FTIR spectrometer to examine the interactions among the constituent components. Each spectrum was obtained by averaging 24 scans over the wavenumber range of 400–4000 cm^−1^.

#### 2.5.3. Thermogravimetric Analysis (TGA)

Thermal degradation behavior of the nanofibrous mats was investigated using a thermogravimetric analyzer (STA 7300, Hitachi, Tokyo, Japan). Samples were heated from room temperature to 600 °C at a constant rate of 10 °C/min under a nitrogen atmosphere with a steady gas flow of 2 mL/min.

#### 2.5.4. XRD

X-ray diffraction (XRD) measurements were carried out at room temperature using a RIGAKU ULTRA IV diffractometer over a Bragg angle (2θ) range of 10–90°. The X-ray source was operated at 30 kV and 20 mA, and data were collected with a step size of 0.03°.

#### 2.5.5. Mechanical Properties

Uniaxial tensile properties of the nanofibrous mats were evaluated using rectangular specimens carefully cut from the electro-spun membranes. The samples had approximate dimensions of 10 mm in width and 50 mm in length, with an effective gauge length of 30 mm between the grips during testing. Specimen thickness was measured using a digital micrometer, and the average value was used for stress calculations. Tensile strength, elongation at break, and maximum load capacity were evaluated using a Shimadzu universal testing machine equipped with a 1 kN load cell. All nanofiber samples containing different concentrations of BCNWs were tested, and the measurements were conducted at a crosshead speed of 1 mm/min.

#### 2.5.6. Water Solubility Test

A water-solubility test was conducted to evaluate the nanofibrous mats’ resistance to dissolution and assess their structural stability under humid conditions. Pre-dried 2 × 2 cm samples were weighed to obtain the initial mass (W_0_) and then immersed in distilled water for 24 h, with gentle stirring as needed. After immersion, the remaining undissolved material was carefully recovered, rinsed with distilled water, and dried at room temperature to a constant weight before measuring final mass (W_f_). Percentage solubility of the nanofiber mats was calculated following the procedure reported by Prus-Walendziak and Kozlowska [24].

(1) $$Water\;solubility\;\left(\%\right)=\left(\frac{W_{0}-W_{f}}{W_{0}}\right)\times 100$$

#### 2.5.7. Water Contact Angle

Surface hydrophobicity of the samples was evaluated by measuring the water contact angle using the sessile drop method at room temperature. A droplet of approximately 0.0085 mL of distilled water was gently placed on the sample surface, and the contact angle was recorded using a video-based contact angle measurement system (Theta Lite).

#### 2.5.8. Swelling Behavior

The swelling behavior of the nanofibrous mats was evaluated to determine their water-interaction capacity and dimensional stability under aqueous conditions. Pre-dried samples with dimensions of 2 × 2 cm were weighed to obtain the initial dry weight (W_0_). The samples were then immersed in distilled water at room temperature for a predetermined period. After immersion, the swollen samples were carefully removed, gently blotted with filter paper to eliminate excess surface water, and immediately weighed to determine the swollen weight (Ws). The swelling ratio was calculated using the following equation:

Swelling ratio (%) = [(Ws − W_0_)/W_0_] × 100

#### 2.5.9. Oil Resistance

Oil/grease resistance of the nanofibrous mats was evaluated using a gravimetric oil uptake method. Pre-dried samples with dimensions of 2 × 2 cm were weighed to determine their initial dry weight (W_0_). Each sample was exposed to vegetable oil at room temperature for a fixed period. After exposure, the samples were removed from the oil, and excess surface oil was gently wiped using filter paper without damaging the fibrous structure. The samples were then weighed immediately to obtain the final weight after oil exposure (Wo). Oil uptake was calculated according to the following equation:

Oil uptake (%) = [(Wo − W_0_)/W_0_] × 100

### 2.6. Statistical Analysis

Unless otherwise stated, all experiments were performed in triplicate. The experimental data were analyzed using analysis of variance (ANOVA) and are reported as mean ± standard deviation. Statistically significant differences among the results were determined using the Duncan post hoc test at a confidence level of *p* < 0.05.

## 3. Results

### 3.1. Morphology of BCNW

**Figure 2:**
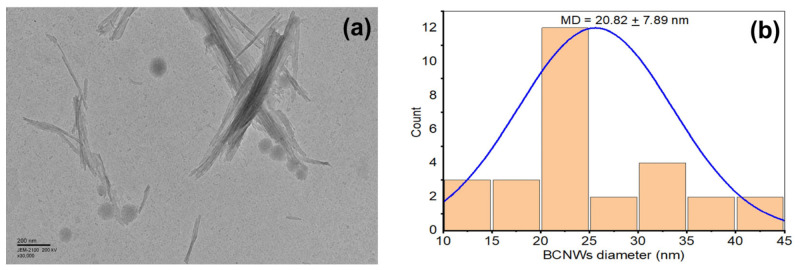
TEM micrograph (**a**) and diameter distribution (**b**) of kombucha-based BCNW.

The morphology of the BCNW was characterized by transmission electron microscopy (TEM). A dilute aqueous suspension of BCNW was sonicated to promote homogeneous dispersion, and a droplet of the suspension was deposited onto a carbon-coated copper grid and dried at room temperature prior to imaging. The TEM micrographs were used to assess the morphology and dimensions of the nanowhiskers. As illustrated in Figure 2, sulfuric acid hydrolysis successfully generated nanoscale cellulose whiskers exhibiting a characteristic needle-like morphology with high aspect ratios, confirming the effective conversion of bacterial cellulose into nanowhisker structures. As described in the BCNW synthesis section, the nanowhiskers were dispersed in deionized water. However, Figure 2a reveals localized agglomeration and noticeable variability in whisker lengths, which are typical characteristics of cellulose nanowhiskers arising from their strong intermolecular interactions. The needle-like morphology indicates that sulfuric acid treatment effectively removed amorphous regions while retaining the crystalline domains of cellulose [25]. Image analysis via Image J software showed that the BCNW had a diameter of 20.82 + 7.89 nm, within the range commonly reported for bacterial cellulose nanowhiskers (10–30 nm) [26,27]. BCNWs clearly have a high aspect ratio, a key feature for efficient reinforcement in polymer matrices. These nanoscale characteristics promote effective stress transfer and are expected to enhance mechanical strength and barrier performance in the resulting composite nanofibers.

### 3.2. XRD Results of BCNW

**Figure 3:**
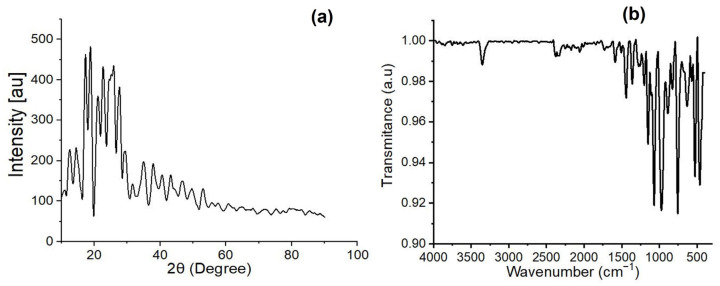
XRD (**a**) and FTIR (**b**) spectra of BCNW.

The crystalline structure of the prepared bacterial cellulose nanowhiskers (BCNWs) was investigated by X-ray diffraction (XRD), and the obtained diffraction pattern is presented in Figure 3a. The XRD profile exhibited the characteristic diffraction peaks of native cellulose I, with reflections located at approximately 2θ = 14.6°, 16.8°, 22.6°, and 34.5°, corresponding to the (1–10), (110), (200), and (004) crystallographic planes, respectively. These characteristic reflections confirm that the crystalline structure of bacterial cellulose was preserved after sulfuric acid hydrolysis, which is consistent with the diffraction patterns reported for cellulose I in the literature [28,29].

No additional diffraction peaks were observed after hydrolysis, indicating that the sulfuric acid treatment did not induce a polymorphic transformation of cellulose. Instead, the acid selectively hydrolyzed the less ordered amorphous regions while preserving the highly ordered crystalline domains, resulting in the formation of cellulose nanowhiskers. This selective removal of amorphous cellulose during sulfuric acid hydrolysis has been widely reported and is considered the primary mechanism for producing cellulose nanocrystals with high crystallinity [30].

Furthermore, the relatively sharp and intense diffraction peak corresponding to the (200) crystallographic plane suggests that the obtained BCNWs possess a high degree of crystallinity. High crystallinity is a characteristic feature of bacterial cellulose and is directly associated with its excellent mechanical strength, stiffness, and reinforcing capability when incorporated into polymer matrices [31]. The XRD results are also consistent with the SEM observations, where the hydrolyzed cellulose exhibited a needle-like morphology typical of cellulose nanowhiskers. Together, these findings confirm that sulfuric acid hydrolysis successfully produced BCNWs while preserving the cellulose I crystalline structure, thereby providing direct experimental evidence that supports the morphological observations.

### 3.3. FTIR Results of BCNW

The chemical structure and functional groups of the prepared bacterial cellulose nanowhiskers (BCNWs) were characterized using Fourier-transform infrared (FTIR) spectroscopy, and the resulting FTIR spectrum is presented in Figure 3b. The FTIR spectrum of bacterial cellulose nanowhiskers exhibited characteristic cellulose absorption bands, confirming the preservation of the cellulose backbone after nanowhisker extraction. The broad absorption band observed at approximately 3340 cm^−1^ corresponds to O–H stretching vibrations associated with intermolecular hydrogen bonding. The peak at 2895 cm^−1^ is attributed to C–H stretching vibrations of aliphatic groups. The band near 1640 cm^−1^ indicates the bending vibration of absorbed water molecules. The peaks at 1430 and 1370 cm^−1^ correspond to CH_2_ bending and C–H deformation, respectively, reflecting the crystalline nature of cellulose. The strong bands at 1162 and 1058 cm^−1^ are assigned to C–O–C glycosidic bond stretching and C–O stretching vibrations. The peak around 897 cm^−1^ confirms the β-glycosidic linkages characteristic of cellulose I. These results indicate that the nanowhisker preparation process did not significantly alter the chemical structure of bacterial cellulose.

### 3.4. Characterization of Nanofibrous Mats

#### 3.4.1. SEM

**Figure 4:**
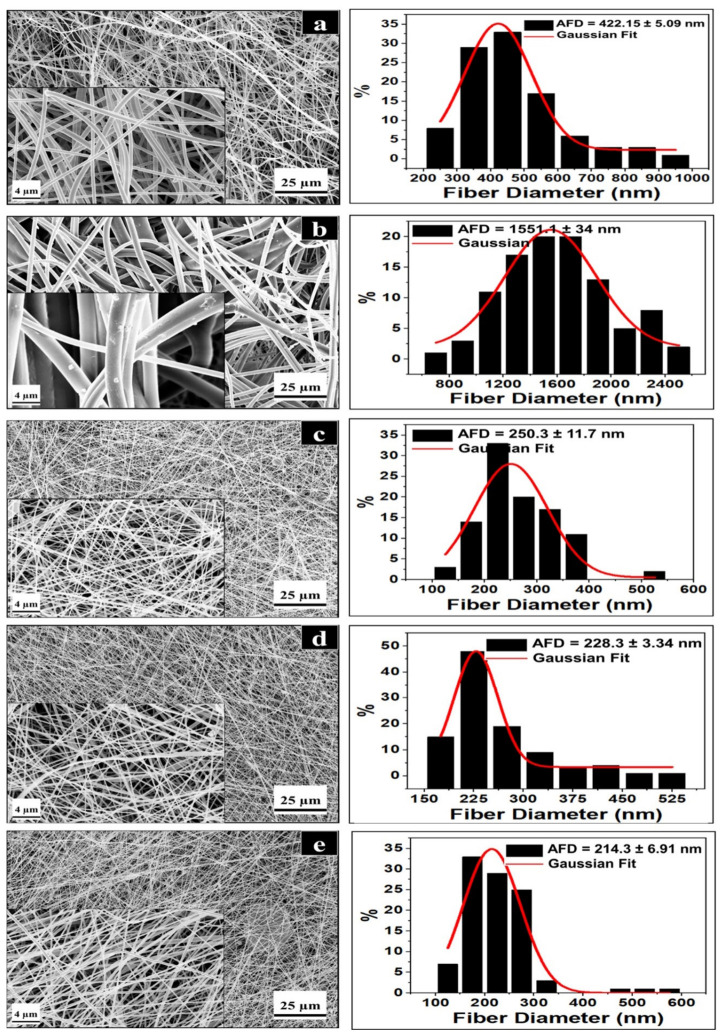
SEM images of G-PVA (**a**), G-PVA-2BCNW (**b**), G-PVA-4BCNW (**c**), G-PVA-6BCNW (**d**), and G-PVA-10BCNW (**e**) nanofibrous mats.

Figure 4 shows the morphological features of the nanofibrous G-PVA mats, confirming the successful formation of nanoscale fibers. The red line in the distribution function graphs represents a Gaussian fit of the fiber diameter distribution. Overall, all samples consisted predominantly of uniform nanofibers with minimal bead formation and occasional fiber bundles, indicating stable fiber formation during electroblowing. Notably, no droplets were observed in any of the fabricated mats. The formation of beads and fiber bundles is likely associated with air turbulence during the electroblowing process [25,26]. The G-PVA nanofiber mat had an average fiber bundle diameter of 422.15 ± 5.09 nm. Adding 2 wt.% BCNW to the G-PVA matrix (referred to as G-PVA-2BCNW) yielded a comparable fiber diameter of 404.58 ± 16.94 nm, indicating that this low concentration of BCNW had no significant effect on fiber thickness. However, increasing the BCNW concentration to 4, 6, and 10 wt.% led to a notable reduction in fiber diameter, with mean diameters of 250.3 ± 11.7 nm, 228.3 ± 3.34 nm, and 214.3 ± 6.91 nm, respectively.

The reduction in the average fiber diameter with increasing BCNW content is likely associated with changes in the solution properties induced by the incorporation of nanowhiskers. Previous studies have shown that parameters such as electrical conductivity, viscosity, surface tension, and polymer–nanofiller interactions collectively influence jet stretching and the resulting fiber diameter during electrospinning and electroblowing [32,33]. Since the electrical conductivity of the spinning solutions was not measured in the present study, its contribution cannot be confirmed experimentally. Therefore, the observed decrease in fiber diameter is attributed to the combined influence of these factors rather than to conductivity alone [34]. Cellulose nanowhisker, which are highly charged nanostructures due to carboxyl functional groups, significantly enhances the solution’s ionic conductivity [35]. The increased availability of charge carriers and enhanced charge density promote the development of stronger electrostatic forces within the electrospinning jet, reducing diameter size.

#### 3.4.2. FTIR

**Figure 5:**
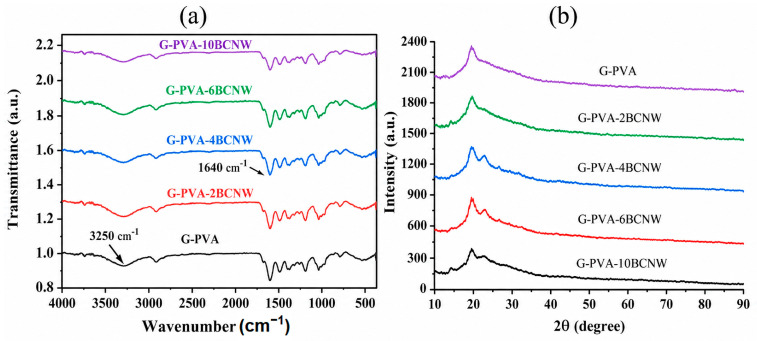
FTIR spectra (**a**) and XRD (**b**) results of nanofiber mats.

FTIR was used to investigate potential physical and chemical interactions among the functional groups of G’s, PVA, and BCNW, as shown in Figure 5a. Nanofibrous webs exhibited peaks at 1087.12 cm^−1^ (-C-C-O), 1242 cm^−1^ (amide-III), 1427 cm^−1^ (-CH2 bending), 1530 cm^−1^ (amide-II), 1640 cm^−1^ (amide-I), 2920 cm^−1^ (CH2 asymmetric stretching), and 3296 cm^−1^ (-OH stretching) [29,30]. In all nanofibrous webs, the 3296 cm^−1^ peak was more distinct than the other characteristic peaks. For the protein (gelatin) component, the characteristic amide I (C=O stretching, ɤC=O), amide II (N–H bending, δN–H), and amide III (C–N stretching, ɤC–N) bands were observed at approximately 1650, 1530, and 1230 cm^−1^, respectively [36]. Incorporating BCNW into the G-PVA matrix did not produce significant changes in the observed peaks, either in position or intensity. The absence of notable peak shifts after adding BCNW to G-PVA suggests that little or no chemical reactions occurred among the functional groups; however, inter- or intramolecular bonding may still have occurred.

#### 3.4.3. XRD

As shown in Figure 5b, the XRD pattern of the neat G–PVA nanofibrous mat exhibits a broad diffraction feature centered at approximately 2θ = 20°, which is characteristic of materials containing a substantial amorphous component. Similar diffraction behavior has been reported for gelatin-based systems, where broad peaks near 20° are associated with the disordered arrangement of polymer chains. Upon incorporation of BCNW (2, 4, 6, and 10 wt.%), noticeable changes in the shape and relative prominence of this broad diffraction feature were observed, suggesting that the nanowhisker influences the molecular organization and packing of the gelatin–PVA matrix. These changes are likely related to intermolecular interactions between BCNW and the polymer chains, which can alter chain arrangement and structural ordering within the matrix. Comparable observations were reported by Bai et al. [37], who observed that the addition of cellulose nanowhisker to polymer matrices disrupts the orderly arrangement of polymer chains, thereby decreasing crystallinity.

Although BCNW is known to possess semi-crystalline characteristics with diffraction peaks typically appearing near 14°, 16°, and 22°, these peaks were not distinctly observed in the composite samples. This may be attributed to the relatively low BCNW content and the dominance of the broad diffraction feature associated with the gelatin–PVA matrix [36]. The observed changes in the diffraction patterns suggest an increase in the amorphous character of the gelatin–PVA matrix following BCNW incorporation. Such structural modifications can influence the mechanical, thermal, and barrier properties of the material by altering polymer chain mobility and intermolecular interactions. Similar behavior has been reported by Yu Juan et al. [38], who demonstrated that a decrease in matrix crystallinity in polymer composites can lead to enhanced flexibility and greater water uptake compared with the corresponding unfilled polymer.

#### 3.4.4. Thermal Stability Analysis

**Figure 6:**
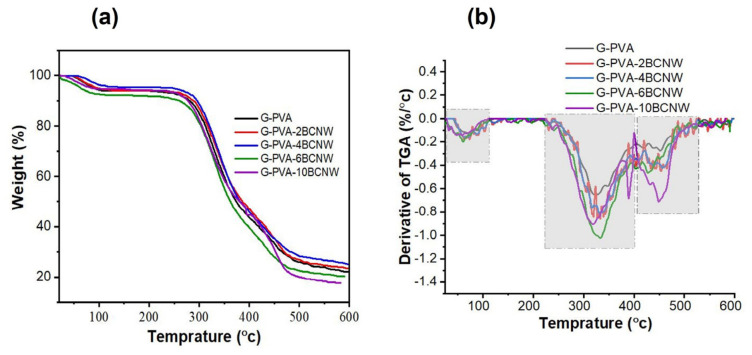
TGA (**a**) and DTGA (**b**) results for nanofiber mats.

TGA profiles in Figure 6 exhibited three distinct stages of thermal degradation. The initial minor mass loss of ≈10% was observed at 60–110 °C due to the evaporation of physically adsorbed and bound moisture in the hydrophilic gelatin–PVA matrix [39]. The second major degradation stage occurred in the range of 300–450 °C due to the thermal decomposition of the polymer backbone, including the degradation of gelatin peptide structures and the elimination reactions along the PVA chains corresponding to the thermal decomposition of the cellulose and PVA matrix [40]. A final degradation stage at higher temperatures corresponds to the breakdown of the remaining polymeric residues and carbonaceous structures.

The incorporation of BCNW into the G–PVA matrix influenced its thermal degradation behavior. To quantitatively compare thermal stability, the temperature corresponding to 50% weight loss (T_50_) was evaluated for all samples. Among the investigated samples, the G–PVA composite containing 4 wt.% BCNW exhibited the highest T_50_ value, which was 371.49 ± 17.4 °C, indicating improved thermal stability relative to the other compositions, which correlates well with the highest crystallinity observed in Figure 4b. However, a direct correlation between thermal stability and crystallinity requires quantitative crystallinity analysis through XRD peak deconvolution and curve fitting. An improved crystalline structure is known to contribute to increased thermal resistance and elevated degradation temperatures [41], which is consistent with previous reports [42]. The improved thermal stability is attributed to strong interfacial interactions between the CNFs and the PVA matrix, which limit polymer chain mobility and thereby reduce mass loss during thermal degradation [43].

Furthermore, the presence of extended nanocellulose crystal chains, interconnected by hydrogen bonding and arranged in an ordered manner, contributed to the improved thermal resistance of the samples [43]. These findings are consistent with those reported by Yihun et al. [44]. Upon further heating beyond 435 °C, a third degradation step was observed, associated with the decomposition of residual ash [41].

The characteristic thermal degradation parameters obtained from the TGA/DTG curves are summarized in Table 1. The table includes the temperatures corresponding to the maximum moisture loss, the main and secondary degradation stages, and the residual mass remaining at 600 °C for each sample.

#### 3.4.5. Mechanical Property Analysis

**Figure 7:**
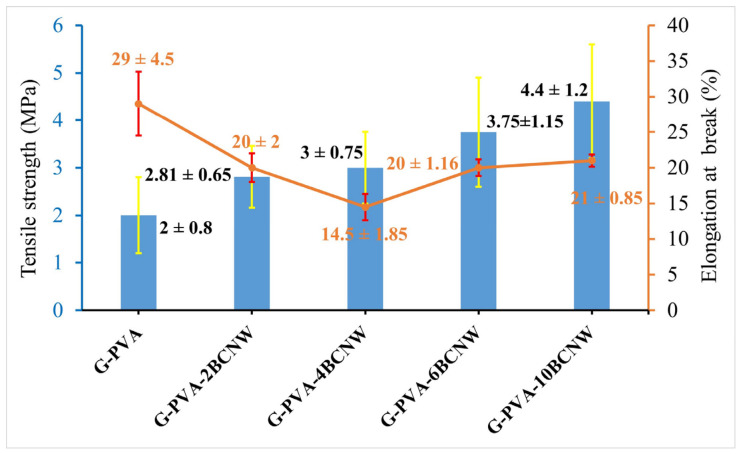
Mechanical test results of all nanofibrous mats.

Mechanical test results shown in Figure 7 reveal that incorporating BCNW into the G-PVA matrix significantly influences both tensile strength and elongation at break. The tensile strength of neat G-PVA was measured at 2 ± 0.8 MPa and increased progressively with the addition of BCNW, reaching a maximum of 4.4 ± 1.2 MPa at 10% loading. This notable enhancement suggests that BCNWs effectively reinforce the polymer network by promoting strong interfacial interactions and stress transfer, a phenomenon consistent with reports by Dagang Liu et al. [45], who demonstrated that cellulose nanowhiskers improve tensile strength in PVA-based nanocomposites by forming hydrogen bonds and restricting chain mobility. However, elongation at break exhibited an inverse trend; the addition of BCNWs initially reduced flexibility, decreasing from 29 ±4.5% in neat G-PVA to 14.5 ± 1.85% at 4% BCNW, indicating restricted polymer chain movement, as similarly observed by Melbi Mahardika et al. [46], who reported that nanocellulose fillers reduce ductility due to stiff filler domains. Interestingly, higher BCNW contents (6% and 10%) resulted in a partial recovery of elongation to around 20–21%, which may be attributed to the formation of a percolated nanofiller network that balances stiffness and ductility, in line with findings by Takaaki Kasuga [47], who noted that higher nanocellulose loadings can create interconnected structures that partially restore deformability. Overall, these findings demonstrate a clear trade-off between tensile strength and elongation, underscoring the importance of optimizing BCNW concentration to achieve desirable mechanical performance for specific applications.

#### 3.4.6. Water Contact Angle

**Figure 8:**
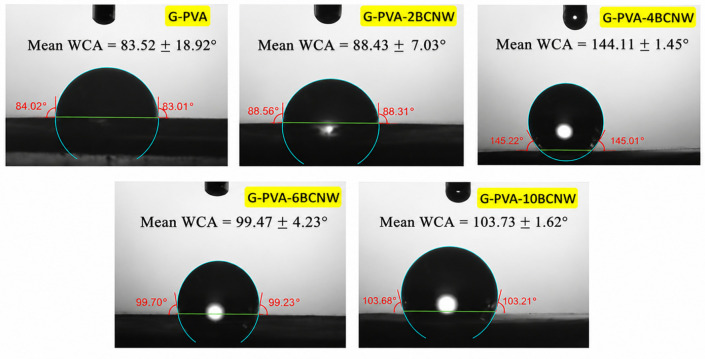
Average water contact angle (WCA) of all nanofiber mats.

Water contact angle (WCA) measurements, shown in Figure 8, were conducted to evaluate the surface wettability of the G-PVA and G-PVA-BCNW composite mats. Surface wettability plays a key role in material performance, with higher contact angles indicating greater hydrophobicity and lower values reflecting hydrophilic behavior. The pure G-PVA sample exhibits a WCA of 83.52 ± 18.92°, confirming its hydrophilic nature, which arises from the abundant hydroxyl (-OH) and amide (-CONH) functional groups present in both gelatin and polyvinyl alcohol [48].

The incorporation of BCNW resulted in variations in the water contact angle (WCA); however, a strictly consistent trend was not observed. This behavior is expected for electro-spun nanofibrous mats, where surface roughness, porosity, and random fiber arrangement significantly influence wettability measurements. All samples except the 2% BCNW sample exhibited hydrophobic behavior (WCA > 90°), which is desirable for food packaging applications. Among the tested compositions, the 4% BCNW sample showed the highest WCA, indicating the most pronounced hydrophobic surface. The samples containing 6% and 10% BCNW exhibited lesser hydrophobicity, while the 2% BCNW sample was slightly below the hydrophobic–hydrophilic threshold.

The enhanced hydrophobicity observed in certain samples, particularly at 4 wt% BCNW loading, may plausibly be attributed to changes in the surface morphology induced by BCNW incorporation. As evidenced by the SEM images, the incorporation of BCNWs resulted in finer fibers and a more compact fibrous network, which may have increased surface roughness and promoted the formation of hierarchical surface features. Such morphological changes have been reported to reduce wettability by increasing the apparent contact angle. Similar observations were reported by Wang et al. [49] and Sun Lin et al. [50], who suggested that cellulose nanowhisker-induced hierarchical structures can enhance hydrophobic behavior through increased surface roughness.

In contrast, increasing the BCNW content to 10 wt% resulted in a decrease in the water contact angle (103.73 ± 1.62°). This behavior may be associated with partial aggregation of BCNWs at higher loadings, as suggested by the SEM observations, which could reduce surface uniformity and increase the exposure of the inherently hydrophilic hydroxyl groups present on the nanowhiskers. A similar trend was reported by Wardhono et al. [51], who found that excessive nanocellulose incorporation adversely affected the surface wettability of polymer composites. Considering the heterogeneous nature of electroblown nanofibrous mats, localized variations in fiber packing density, surface roughness, and nanowhisker distribution may also contribute to the observed differences in water contact angle. Therefore, these variations are more appropriately interpreted as plausible effects of morphological changes rather than as evidence of a strictly non-monotonic relationship between BCNW content and wettability.

Overall, the results indicate that BCNW incorporation influences the surface wettability of G–PVA nanofibrous mats, with the observed changes being reasonably explained by the combined effects of fiber morphology and nanowhisker distribution. Among the investigated compositions, the sample containing 4 wt% BCNW exhibited the highest water contact angle, suggesting that this loading provided the most favorable surface morphology for enhancing hydrophobicity under the present experimental conditions.

#### 3.4.7. Water Solubility

**Figure 9:**
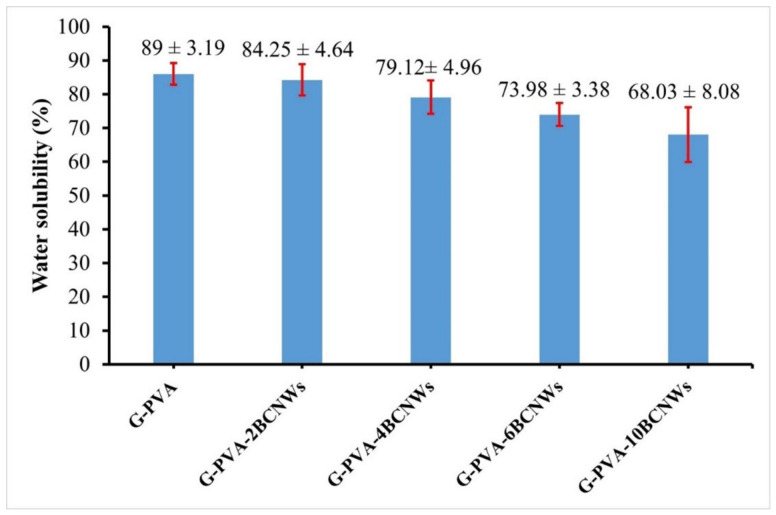
Water-solubility test results for all nanofibrous mats.

A decrease in water solubility, as shown in Figure 9, was observed with increasing BCNW content in the G-PVA system. The decrease in solubility can be explained by several complementary mechanisms. First, strong interfacial hydrogen bonding between the hydroxyl-rich surface of BCNW and polymer chains reduces the availability of free hydrophilic groups capable of interacting with water [52]. Second, the highly crystalline and rigid nature of BCNW increases the overall network density and crystallinity of the composite matrix, thereby limiting polymer chain mobility and reducing susceptibility to dissolution [53]. Third, uniformly dispersed nanowhiskers create a more tortuous pathway for water penetration, slowing diffusion into the fibrous network [54].

The relatively higher standard deviation observed at 10% BCNW (±8.08%) likely reflects the onset of BCNW aggregation or increased microstructural heterogeneity at elevated loadings. This observation is consistent with previous studies that emphasize the critical role of nanofiller dispersion in achieving consistent water-resistance performance. While BCNW incorporation markedly improves water resistance, the maintained partial solubility (~68%) indicates that additional modifications, such as surface treatment of BCNWs, polymer crosslinking, or the application of a hydrophobic coating, may be necessary for applications requiring near-impermeability [55].

The variation in water solubility with increasing BCNW content can be attributed to the influence of the nanowhisker on the structural integrity of the gelatin–PVA matrix. The incorporation of BCNWs disrupts the continuity of the polymer network and facilitates water penetration. Moreover, the hydrophilic nature of BCNWs, due to the abundance of hydroxyl groups, enhances the interaction with water molecules. While hydrogen bonding between BCNWs and the polymer matrix may provide partial stabilization at lower loadings, higher BCNW contents and possible nanowhisker aggregation can lead to increased solubility. These findings are consistent with previous reports on nanocellulose-reinforced polymer systems by Punia H. et al. [56] and Kunnath SM. et al. [57].

Although water absorption (swelling) analysis can provide complementary information regarding the interaction of the material with water, water solubility was considered the most relevant parameter for evaluating the moisture resistance of the nanofibrous mats in food packaging applications. Therefore, water absorption testing was not included in the present study but will be considered in future work.

#### 3.4.8. Swelling Behavior and Oil Resistance

The swelling behavior and oil uptake capacity of the G-PVA and G-PVA/BCNW nanofibrous mats were evaluated to further assess their water-interaction behavior and resistance against oily media, which are important considerations for food-packaging materials. The results are presented in Table 2. The neat G-PVA nanofibrous mat showed the highest swelling ratio, with a value of 425.6 ± 32.8%, indicating strong affinity toward water. This behavior can be mainly attributed to the hydrophilic nature of both gelatin and PVA, which contain abundant hydroxyl, amide, and other polar functional groups capable of interacting with water molecules. In particular, PVA is known to be highly sensitive to humid or aqueous environments because of the large number of hydroxyl groups along its polymer backbone [58].

The incorporation of BCNWs significantly reduced the swelling ratio of the nanofibrous mats. The swelling ratio decreased from 425.6 ± 32.8% for neat G-PVA to 362.4 ± 27.5% for G-PVA-2BCNW and reached the lowest value of 248.9 ± 21.6% for G-PVA-4BCNW. This corresponds to an approximately 41.5% reduction in swelling compared with the neat G-PVA mat. The reduced swelling at moderate BCNW loading may be attributed to the formation of hydrogen-bonding interactions between the hydroxyl-rich BCNW surface and the gelatin/PVA matrix, which can reduce the availability of free hydrophilic groups and restrict polymer chain mobility. Moreover, the highly crystalline and rigid structure of BCNWs may contribute to a denser and more stable network, limiting excessive water penetration into the fibrous structure. Similar behavior has been reported for PVA/gelatin films reinforced with cellulose nanocrystals, where the addition of nanocellulose decreased water absorption, moisture uptake, and water vapour permeability [59]. The role of nanocellulose–water interactions in controlling the moisture response of nanocellulose-containing materials has also been extensively discussed by Solhi et al. [52]. However, further increasing the BCNW concentration beyond 4 wt.% resulted in a partial increase in swelling ratio. The G-PVA-6BCNW and G-PVA-10BCNW samples showed swelling ratios of 286.7 ± 24.9% and 334.2 ± 36.1%, respectively. Although these values were still lower than that of neat G-PVA, the increase compared with G-PVA-4BCNW suggests that excessive BCNW loading may cause partial nanowhisker aggregation and microstructural heterogeneity. Such aggregation can disrupt the uniformity of the polymer network, generate local defects, and facilitate water penetration into the nanofibrous mat. Therefore, the swelling results indicate that 4 wt.% BCNW provided the most effective improvement in water resistance among the investigated formulations.

A similar trend was observed for oil uptake. The neat G-PVA mat exhibited the highest oil uptake value of 184.3 ± 14.6%, indicating relatively poor resistance to oil penetration. The incorporation of BCNW significantly decreased oil uptake, with values of 151.7 ± 11.3%, 91.8 ± 8.7%, 108.5 ± 9.4%, and 137.6 ± 12.8% for G-PVA-2BCNW, G-PVA-4BCNW, G-PVA-6BCNW, and G-PVA-10BCNW, respectively. The lowest oil uptake was obtained for the G-PVA-4BCNW sample, corresponding to an approximately 50.2% reduction compared with neat G-PVA. This improvement may be associated with the formation of a more compact fibrous network, improved filler dispersion, reduced pore accessibility, and enhanced tortuosity against oil penetration. The improved oil/grease resistance at moderate BCNW loading is consistent with previous reports indicating that nanocellulose-based coatings and composites can act as effective oil and grease barriers by decreasing porosity and forming dense hydrogen-bonded networks [60,61]. Tyagi et al. reported that nanocellulose-based multilayer coatings provided high oil and grease resistance, while Mazega et al. showed that coating formulations containing nanocellulose and PVA could achieve high Kit ratings for grease resistance [60]. In the present study, the increase in oil uptake at 6 and 10 wt.% BCNW may again be related to nanowhisker aggregation and structural heterogeneity at higher filler concentrations. These findings suggest that the incorporation of BCNW, particularly at 4 wt.%, improves both water-interaction behavior and oil/grease resistance of G-PVA nanofibrous mats. Overall, the swelling and oil uptake results demonstrate that BCNW incorporation can enhance the resistance of gelatin/PVA nanofibrous mats against water and oily media. Among the tested formulations, G-PVA-4BCNW showed the most favorable balance, exhibiting the lowest swelling ratio and oil uptake.

## 4. Conclusions

This study successfully demonstrated the bio-reinforcement of electroblown gelatin/polyvinyl alcohol (G-PVA) nanofibrous mats using kombucha-derived cellulose nanowhiskers (BCNWs) as a sustainable strategy to enhance mechanical and surface performance for food packaging applications. The valorization of kombucha symbiotic culture of bacteria and yeast (SCOBY) as a source of high-aspect-ratio cellulose nanowhiskers aligns with circular economy principles while providing an effective reinforcing phase for biodegradable polymer systems.

The addition of BCNWs markedly enhanced the tensile performance of the nanofibrous mats, achieving a maximum increase in tensile strength of 120% at 10 wt.% BCNW. This improvement is attributed to effective stress transfer and strong interfacial interactions between the nanowhiskers and the G–PVA matrix. Morphological analysis confirmed the formation of uniform, bead-free nanofibers at appropriate BCNW loadings, supporting improved mechanical integrity. Structural characterization revealed favorable intermolecular interactions without chemical degradation, while thermal analysis indicated enhanced thermal stability at moderate BCNW contents, with agglomeration effects becoming evident at higher loadings. Furthermore, surface wettability was substantially improved, achieving a maximum water contact angle of 144.11 ± 1.45°, highlighting the role of BCNW in tailoring surface functionality.

Overall, the developed BCNW-reinforced electroblown gelatin/PVA nanofibrous mats demonstrated improved mechanical performance, enhanced surface wettability characteristics, and reduced water solubility, indicating their potential as sustainable bio-based materials. However, comprehensive evaluations of packaging-specific properties, including water vapor and gas barrier performance, migration behavior, food preservation efficiency, and long-term stability, are required before their practical application can be established. Therefore, the developed nanofibrous mats are proposed as promising candidate materials for sustainable food packaging, warranting further investigation.

Although the developed BCNW-reinforced G–PVA nanofibrous mats exhibited improved morphological, mechanical, and wettability properties, several limitations should be acknowledged. The proposed mechanisms governing the wettability changes are based on the observed fiber morphology and previously reported findings rather than direct surface characterization. Therefore, future studies should include surface roughness and surface chemical analyses to establish direct correlations between BCNW distribution, surface characteristics, and wettability. In addition, systematic characterization of the electroblowing solution properties, including viscosity, electrical conductivity, surface tension, pH, and processing conditions, is needed to better understand their influence on fiber formation. Furthermore, comprehensive evaluation of barrier properties (water vapor and oxygen permeability), migration behavior, biodegradability, and real food-storage performance should be conducted to validate the suitability of the developed nanofibrous mats for practical food packaging applications.

## Figures and Tables

**Table 1:** Summary of (TGA) and derivative thermogravimetric (DTG) parameters of all nanofibrous mats.

Sample	Moisture Loss Tmax (°C)	Main Degradation Tmax (°C)	Secondary Degradation Tmax (°C)	Residual Mass at 600 °C (%)
G-PVA	73.13 ± 17.45	327.09 ± 25.62	452.55 ± 10.64	22.17
G-PVA-2BCNW	71.13 ± 7.85	332.66 ± 18.95	465.06 ± 9.75	23.63
G-PVA-4BCNW	87.47 ± 12.61	336.13 ± 14.76	455.89 ± 16.72	25.38
G-PVA-6BCNW	61.63 ± 5.27	335.03 ± 12.95	427.77 ± 13.45	20.28
G-PVA-10BCNW	60.59 ± 23.34	321.33 ± 9.17	448.59 ± 28.96	17.62

**Table 2:** Swelling behavior and oil resistance of nanofibrous mats.

Sample Code	Swelling Ratio (%)	Oil Uptake (%)
G-PVA	425.6 ± 32.8ᵃ	184.3 ± 14.6ᵃ
G-PVA-2BCNW	362.4 ± 27.5ᵇ	151.7 ± 11.3ᵇ
G-PVA-4BCNW	248.9 ± 21.6ᵈ	91.8 ± 8.7ᵈ
G-PVA-6BCNW	286.7 ± 24.9ᶜᵈ	108.5 ± 9.4ᶜᵈ
G-PVA-10BCNW	334.2 ± 36.1ᵇᶜ	137.6 ± 12.8ᵇᶜ

## Data Availability

The data presented in this study are available from the corresponding author upon reasonable request.

## Abbreviations

The following abbreviations are used in this manuscript:

G	Gelatin
PVA	Poly (vinyl alcohol)
BCNW	Bacterial cellulose nanowhisker
SCOPY	Symbiotic culture of bacteria and yeast
TGA	Thermogravimetric Analysis
FTIR	Fourier Transform Infrared Spectroscopy
XRD	X-ray Diffraction
WCA	Water Contact Angle
Wt.%	Weight percent
